# Interleukin-22 in Renal Protection and Its Pathological Role in Kidney Diseases

**DOI:** 10.3389/fimmu.2022.851818

**Published:** 2022-03-31

**Authors:** Qianqian Ma, Jingyun Luan, Yu Bai, Caili Xu, Fangyu Liu, Bufeng Chen, Dianwen Ju, Hong Xu

**Affiliations:** ^1^ Children’s Hospital of Fudan University, National Children’s Medical Center, Shanghai, China; ^2^ Department of Biological Medicines, School of Pharmacy, Fudan University, Shanghai, China; ^3^ Shanghai Engineering Research Center of Immunotherapeutics, School of Pharmacy, Fudan University, Shanghai, China; ^4^ Department of Neurology, The First Affiliated Hospital of Chongqing Medical University, Chongqing, China; ^5^ Department of Urology, Binzhou Medical University, Binzhou, China

**Keywords:** interleukin-22, basic features, biological effects, the protective role, the pathological effect, kidney diseases

## Abstract

Chronic kidney injury has gradually become a worldwide public health problem currently affecting approximately 10% of the population and can eventually progress to chronic end-stage renal disease characteristic by the result of epithelial atrophy. Interleukin-22 (IL-22) is a cytokine produced by activated immune cells, while acting mainly on epithelial cells ranging from innate immune response to tissue regeneration to maintain barrier integrity and promote wound healing. Accumulating data suggests that IL-22 has emerged as a fundamental mediator of epithelial homeostasis in the kidney through promoting tissue repair and regeneration, inhibiting oxidative stress, and producing antimicrobial peptides. Binding of IL-22 to its transmembrane receptor complex triggers janus kinase/tyrosine kinase 2 phosphorylation, which further activates a number of downstream cascades, including signal transducer and activator of transcription 3, MAP kinase, and protein kinase B, and initiates a wide array of downstream effects. However, the activation of the IL-22 signaling pathways promotes the activation of complement systems and enhances the infiltration of chemokines, which does harm to the kidney and may finally result in chronic renal failure of different autoimmune kidney diseases, including lupus nephritis, and IgA nephropathy. This review describes current knowledge of the basic features of IL-22, including structure, cellular origin and associated signaling pathways. Also, we summarize the latest progress in understanding the physiological and pathological effects of IL-22 in the kidney, suggesting the potential strategies for the specific application of this cytokine in the treatment of kidney disease.

## Introduction

The immune system is developed to guard the host against a variety of pathogens while preventing collateral injury and promoting regeneration, wound healing and tissue repair (1). A large proportion of these functions are closely related to the role of cytokines produced by immune cells and functioning through autocrine and paracrine mechanisms (2). Interleukin-22 (IL-22), known as a cytokine, can be produced immediately after injury and initiates an immune response against several types of tissue impairments (3). Studies have exhibited that IL-22 is a critical signaling molecule that exerts multiple biological effects, including antimicrobial immunity, tissue regeneration, inhibition of oxidative stress, activation of complement systems and recruitment of chemokines (3). However, the beneficial and harmful effects of IL-22 may vary among different disease states (4). It is imperative to clarify the specific pathways and mechanisms by which IL-22 transforms between normal physiological processes and pathological states.

The kidney is one of the most complicated organs physiologically, structurally, and metabolically (5). Renal impairment has affected more than 10% of the population and contributes to high morbidity and mortality. Current treatments for kidney disease are mainly immunosuppressive agents, antihypertensives and diuretics, but the effectiveness of which in the clinic is limited, mainly because of their tendency to cause electrolyte disorders, impede growth, microbial infections, drug dependence and other side effects (6). When exploring new treatments, the physiological heterogeneity of the kidneys should be taken into consideration, such as cellular energy metabolism, hypoxia signaling pathways, renal tissue oxygenation and autoimmunity. IL-22 has been reported to be involved in many of these physiological and pathological processes (7). In the pathophysiology of acute kidney injury (AKI), hypoxia-inducible factor (HIF-1α) is expressed in renal tubular cells and can increase the secretion of IL-22 through the activation of CD4^+^ T cells, thereby regulating glucose metabolism and increasing glycogen storage (8). In addition, IL-22 ameliorates mitochondrial dysfunction and reactive oxygen radical production and inhibits the activation of the NOD-like receptor family containing protein 3 (NLRP3) inflammasome, which consequently reduces inflammatory responses (9, 10). On top of that, IL-22 acts as a regenerative survival factor in renal tissues (11). When the proximal tubules and renal cortical cells in the extrarenal medullary strip are damaged, damage-associated molecular patterns (DAMPs) are released. Dying renal tubular epithelial cells (RTECs) recruit mesenchymal dendritic cells to release IL-22. Toll-like receptor 4 (TLR4)-IL-22 pathway and downstream signaling including signal transducer and activator of transcription 3 (STAT3)/protein kinase B (AKT) are consequently activated to reduce apoptosis and promote regeneration of TECs (12). Furthermore, the effect of IL-22 on the regulation of autoimmune kidney disease is another important area of research. The kidney is acted as a vital objective of pathogenic immune responses against autoantigens or systemic autoimmunity (1). Activation of complement systems and infiltration of chemokines promoted by IL-22 during lupus nephritis (LN) and IgA nephropathy (IgAN) are important causes of kidney damage (13, 14).

**Figure 1:**
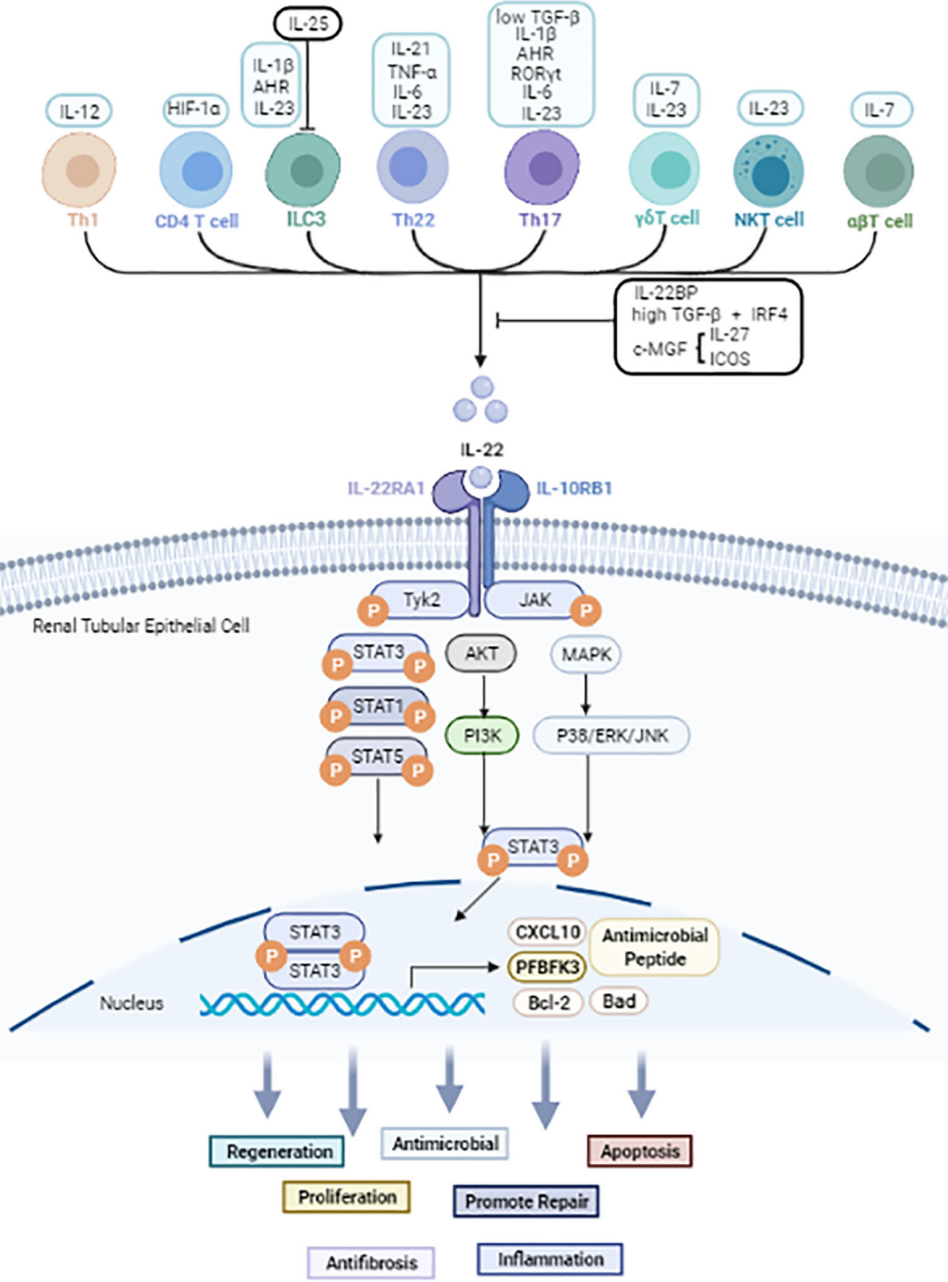
The cellular sources and signals downstream of IL-22 based on current evidence. Black arrow: upregulate. Black line: inhibit. IL-22 is secreted mainly by immune cells. JAK1 and TYK2 molecules are firstly phosphorylated after IL-22 binds to the IL-22R complex. Then further phosphorylate STAT molecules thus causes dimerization and accompanying translocation of STATs to the nucleus. Usually, STAT3 molecules play a pivotal effect, but STAT1 and STAT5 also be involved. Other than JAK/STAT activation, IL-22 also can activate AKT/PI3K and MAPK/P38/ERK/JNK pathways. IL-22 promotes the transcription of proteins such as Bal2, CXCL10 and PFBFK3, and has biological effects including anti-inflammatory, anti-microbial, apoptotic, anti-fibrotic, and proliferative effects in kidney, liver, gastrointestinal tract, and pancreas.

Our understanding of the mechanistic link between IL-22 and kidney disease has improved dramatically in recent years. Considerable data has connected the functions of IL-22 to the pathophysiology underlying the most common kidney diseases. This review will introduce the key structural and biological features of IL-22, and focus on the advances in elucidating the biological functions of the IL-22-IL-22 receptor (IL-22-IL-22R) pathway in kidney-associated infection, tissue repair, oxidative stress and autoimmune kidney injury (**Figure 1**). We will also propose the possibility of targeting IL-22 and its related signaling pathways in the treatment of kidney diseases.

## Interleukin-22: Structure, Sources, and Signaling

### The IL-22 Gene and Protein

IL-22 was initially cloned from BW5147 T-lymphoma cells stimulated by murine IL-9 (15, 16). According to its biochemical and functional characteristics, IL-22 is classified as a class II cytokine, which belongs to IL-10 family (17). Human IL-22 is 79% homologous to mouse IL-22 and 25% identical to human IL-10, initially referred to as “IL-10-related T-cell inducing factor (IL-TIF)” (15). The human *IL-22* gene is located on chromosome 12p15 and contains 5 exons with 5,200 base pairs (18). The secreted human and murine monomeric IL-22 proteins are predicted to be 146 amino acids in length (19). IL-22 is featured with a bundle-like structure consisting of α-helices and connecting loops. There are two disulfide bonds between the N-terminus and the D-E loop, as well as helix C to F forming a stable tertiary structure (19). IL-22 contains three N-glycosylation sites, and the glycosylation may affect the tertiary structure of IL-22 (20). For example, glycosylation of N54 might influence the interaction of IL-22 and IL-10R2 (21). Though the structure change resulting from glycosylation is minor, it should be taken into consideration when using IL-22-specific neutralizing antibodies for treatment (22). Apart from that, the bioactive form of IL-22 appears to be a monomer (23).

### Cellular Sources of IL-22

In general, IL-22 is primarily produced by lymphoid lineage cells, such as T lymphocytes and innate lymphocytes (ILCs) (24). The production of IL-22 in Th1, Th17, and Th22 cells is mainly induced by cytokines such as IL-6, IL-1β, IL-12, IL-21, IL-23, tumor necrosis factor-α (TNF-α) and other molecules such as RORyt and the aryl hydrocarbon receptor (AHR) ligand FICZ (25, 26). In peripheral blood, naïve T lymphocytes can produce IL-22 after differentiation into Th22 cells (27, 28). γδT cells, αβT cells, and NKT cells are capable of producing IL-22 in the presence of IL-7, IL-23, and HIF-1α (24, 29). Apart from adaptive immune cells, ILC3s and NK cells are also key producers of IL-22 (30–32) under stimulation of IL-1β, IL-23 and AhR (33, 34). In addition, the transcription factor c-Maf, inducible costimulatory (ICOS), IL-25, IL-27 and transforming growth factor β (TGF-β) were classified as negative regulators of IL-22 production (35–38).

### Receptors and Intracellular Signals Downstream of IL-22

IL-22R is a heterodimeric complex consisting of IL-22R1 and IL-10R2 subunits, both of which contain an extracellular moiety, an intracellular moiety, and a transmembrane moiety (39). IL-22 alone has a high affinity for the IL-22R1 (Kd values of 1-20 nM) and cannot bind to IL-10R2. However, IL-10R2 has the capability of binding with IL-22-IL-22R1 complex (Kd values of 7-45 μM) (40, 41). Studies have shown that the interaction between IL-22 and IL-22R is a complicated process. Initially, IL-22 binds to IL-22R1 subunit, which leads to a conformational transformation. Then, the IL-22-IL-22R1 complex binds to the IL-10R2 chain. The integrated structure formed by these three parts is capable of eliciting downstream intracellular signal transduction and a wide range of biological effects (24, 41).

IL-22 binding protein (IL-22BP) is known as the other receptor for IL-22, which is composed of 210 amino acids. Its affinity to IL-22 is 20-1000 times higher than that of IL-22R1 (42, 43). Due to its stable combination with IL-22, IL-22BP acts as a natural antagonist of IL-22 that isolates IL-22 from IL-22R1, thus blocking the activation of downstream signaling (44). IL-22BP has been verified to be predominantly expressed in the lung, skin, lymphoid organs, breast, placenta as well as stomach and intestines (45, 46). In addition to the local inhibitory effect, IL-22BP may support the stabilization and systemic functions of IL-22 to some extent.

Upon binding to its receptor complex, IL-22 can activate JAK and TYK2 phosphorylation, which trigger a series of downstream cascade reactions, including activating STAT1, STAT3, and STAT5 phosphorylation (47). Among these effects, the phosphorylation of STAT3 serine residues is necessary for maximal transcriptional activation. Other molecules, including p38, extracellular signal-regulated kinase (ERK), c-Jun N-terminal kinase (JNK), and phosphoinositide 3-kinase (PI3K), are also activated downstream signal pathways mediating the effector function of IL-22 (48–50). IL-22 promotes the transcription of proteins such as Bal2, CXCL10 and PFBFK3, and has biological effects including anti-inflammatory, anti-microbial, apoptotic, anti-fibrotic, and proliferative effects in kidney, liver, gastrointestinal tract, and pancreas.

### The Biological Effects of IL-22

IL-22 is essential in promoting the regeneration of renal tissue and defending against pathogens, but aberrant activation of IL-22 promotes the pathogenesis of various renal diseases (**Table 1**). The dual roles of IL-22 including the protective and the pathological effects in kidney-related diseases are described in detail below.

**Table 1:** Diverse Roles of IL-22 across Different Kidney Diseases.

Kidney diseases	Animal model	*In vitro* system	Major impact	References
**DN**	• Therapeutic plasmid pVAX1mIL22 attenuates metabolic disorders and renal fibrosis in streptozotocin-induced mouse model of DN • IL-22Fc reduces kidney oxidative stress and protects mitochondrial function in db/db Diabetic Nephropathy mice	• Therapeutic plasmid pVAX1mIL22 inhibits ECM accumulation and mesangial matrix expansion in mouse renal glomerular mesangial cells • IL-22Fc stimulates the phosphorylation of STAT3 and AKT and relieves ROS levels in SV40 MES13 cells • IL-22Fc partially alleviates lipid droplets transport in HUVECs	Tissue Regeneration Inhibiting Oxidative Stress	(51, 52)
**AKI**	• IL-22Fc inhibits inflammatory responses and ameliorates renal dysfunction in Acetaminophen-induced kidney injury in mice • IL-22KO mice have a significantly lower survival rate compared with WT mice 7 days after renal I/R • IL-22 treatment or IL-22 overexpression preserves renal function and decreases renal injury after renal I/R in mice	• IL-22Fc reduces the mortality and inhibits oxidative stress of Acetaminophen-induced HK-2 cells • IL-22 treatment reduces renal tubule epithelial cell apoptosis • IL-22–mediated specific activation of STAT3 in the RPTECs prevents RPTEC death	Tissue Regeneration Inhibiting Oxidative Stress	(53, 54)
**CKD**	• Deficiency of IL-22 increases tubular injury in *IL-22^-/-^* compared to *IL-22* **^+/+^** mice in UUO mice model	• rhIL-22 enhances migration, re-epithelialization and barrier function of HK2	Tissue Regeneration	(55)
**IgAN**	• Deficiency of IL-22 or IL-22R alleviates kidney injury in the IgAN model which was induced by administering bovine serum albumin (BSA) in acidified water, CCL4, and castor oil combined with LPS	• None	Aggravating Inflammation	(56)
**LN**	• IL-22 deficiency and IL-22R deficiency alleviate kidney injury in MRL/lpr mice	• rIL-22 upregulates CCL2 and CXCL10 that can recruit macrophages in renal primary epithelial cells and HK2 cells in transwell assay	Aggravating Inflammation	(57, 58)
**The renal complication anemia in CKD**	• Mice deficient in IL-22 or IL-22RA1 develop increases red blood cells (RBCs) and downregulates apoptosis of erythroid precursors. • Exogenous supplementation of rIL-22 exacerbates phenylhydrazine-induced anemia	• IL-22 increases the expression of hepcidin in hepatoma cells	Inducing Apoptosis in Erythroid Precursors	(59–61)

DN, diabetic nephropathy; AKI, acute kidney injury; CKD, chronic kidney diseases; IgAN, IgA nephropathy; LN, lupus nephritis; APAP, acetaminophen; I/R, ischemia/reperfusion; MPO^+^, myeloperoxidase+; IL-22TG, IL-22 overexpression; IL-22KO, IL-22 knockout; ECM, extracellular matrix; ROS, Reactive oxygen species; HUVECs, Human umbilical vein endothelial cells; UUO, unilateral ureteral; RPTECs, renal proximal tube epithelial cells; Hk-2, human proximal tubular cell.

## The Protective Roles of IL-22 in the Kidney

### The Protective Role Against Infections

**Figure 2:**
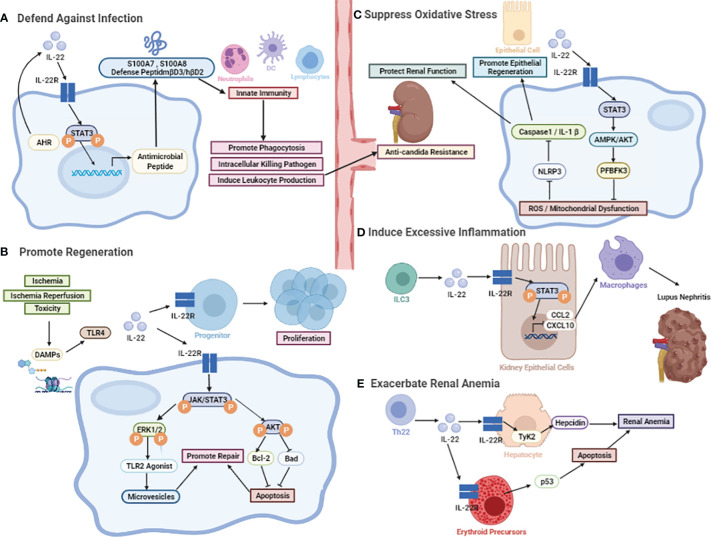
The biological effect of IL-22. **(A)** The protective role against microorganisms including bacterial and fungal. The induction of various antimicrobial peptides, like psoriasis (S100A7), calgranulin-A (S100A8), calgranulin-B (S100A9), β-defensin-3 (mβD3), and lipocalin-2. **(B)** The protective role in promoting regeneration. IL-22-mediated regenerative function by activation of JAK-STAT3 and ERK1/2 phosphorylation. After the onset of AKI, TLR4-IL-22 signaling is a specific stimulus for IL-22 induction. Other than that, activation of STAT3 and AKT can be detected after I/R, followed by an increase in Bcl-2 levels and a decrease in Bad levels. **(C)** The protective role in the inhibition of oxidative stress. It may promote renal repair through the active expression of renal reprogramming PFBFK3 by IL-22 mainly inducing STAT3-AMPK/AKT signaling after injury, slowing down the accumulation of reactive oxygen radical ROS production and mitochondrial dysfunction, which can further protect renal function by inhibiting the NLRP3/Caspase-1/IL-1β signaling pathway. **(D)** The pathological effects in recruiting chemokines. The involvement of IL-22 in the induction of CCL2 and CXCL10, which promotes the infiltration of macrophages to dampen kidney inflammation. **(E)** The pathological effects in inducing chronic renal anemia. IL-22 exacerbates anemia through inducing erythropoietin apoptosis and hepcidin production.

Cytokines are critical contributors in immune response to external challenges and are also particularly important for epithelial barriers (62). In particular, IL-22 is involved in maintaining microflora homeostasis, kidney epithelial cells and a functional defense system against external threats (63) (**Figure 2A**).

The well-documented effect of IL-22 in maintaining microbial homeostasis is mainly achieved by preventing and defending against pathogenic bacterial infections. Production of various antimicrobial peptides, such as psoriasin (S100A7), calgranulin-A (S100A8), calgranulin-B (S100A9), β-defensin-3 (mβD3), and lipocalin-2 serves as a key factor in the IL-22-mediated defense of epithelial cells against pathogenic infection (63). It has been reported that IL-22 plays a major role in protecting skin, gastrointestinal and respiratory tract from pathogenic and commensal bacterial infections through the above mechanisms. Similarly, IL-22 is critical for limiting bacterial replication and hematogenous dissemination in renal infectious diseases, possibly in part by inducing the expression of antimicrobial peptides in epithelial cells on the surface of these barriers. For example, in the case of *Staphylococcus aureus (S. aureus)* infections, the kidney bacterial burden is the basis for invasive or hematogenous dissemination to distant organs (64). Both renal colony-forming units (CFUs) and activated inflammatory factors correlate with disease severity. IL-22 expression was significantly increased at the abscess expression site in the anti-*S. aureus* vaccine NDV-3 injection group compared to the control group, accelerating tissue antimicrobial resistance and repair (65, 66). In contrast, the application of cortisol or neutralizing antibodies to inhibit IL-22 significantly promoted microbial proliferation and the rate of abscess progression in the kidney. Mechanistically, IL-22 may induce the production of host defense peptides to fight against bacterial invasion and protect infected tissues and organs. Both mβD3 in mice and β-defensin-2 in humans increase the adaptive immune response (e.g., dendritic cell or lymphocyte function) while recruiting neutrophils and enhancing phagocytosis and the intracellular killing of pathogens (67, 68). In addition to aiming at *in-situ* infection in the kidney, IL-22 significantly reduces the incidence of hematogenous dissemination after infection, possibly by inducing antimicrobial peptide expression in renal epithelial cells (69). Although little is known whether IL-22 attenuates renal infection through upregulation of related gene expression, IL-22 also acts synergistically with IL-17F or TNF, which stimulates the molecules encoding genes including chemokines CXCL1, CXCL5 and cytokines IL-6 and G-CSF to improve the efficacy of airway, intestinal or skin epithelial cells against bacterial infections (70–72). Furthermore, IL-22 enhances the clearance of abnormal pathogenic microorganisms and regulates flora balance by elevating cytokine and chemokine production in epithelial cells to facilitate leukocyte recruitment and activation at inflammatory sites (73, 74).

IL-22 also exerts a pivotal function in warding off fungal infections of the kidney including primary and hemorrhagic disseminated infections (75). IL-22 is an important natural defender against chronic candidiasis (CMC). As observed in human and mouse models, IL-22 provides the first line of defense against *Candida albicans* by monitoring fungal growth, especially for bloody disseminated kidney fungal infections (69). Studies have reported that tryptophan metabolites from the microbiota balanced mucosal reactivity resist to fungus through promoting the transcription of IL-22 (76). The highly adaptable Lactobacillus expands and produces an AhR ligand that improves AhR-dependent IL-22 transcription and secretion. IL-22 secreted in this way can regulate the imbalance in the flora, allowing for the survival of commensal communities but resisting fungal colonization (69, 76). The effect of IL-22 in defensing against fungi has been reported in pulmonary *Aspergillus fumigatus* infection in lungs and Chronic Mucocutaneous Candidiasis. However, more researches are still needed to find the specific mechanisms by which the kidney protects against fungal infection.

In conclusion, IL-22 is involved in the induction of innate defense mechanisms in renal epithelial tissues, thus protecting tissues from injury during microbial infection and recovery of tissue homeostasis.

### The Protective Role in Tissue Regeneration

Apart from strengthening the host barrier defense against microbial infections, IL-22 is able to stimulate downstream signaling pathways and activate cell survival genes. Increasing evidence suggests that IL-22 is associated with both regenerative and antiapoptotic functions in renal tissues, including acute or chronic kidney injury induced by various etiologies **(** **Figure 2B** **).**

AKI involves an aseptic inflammatory response that exacerbates renal tubular cell damage. Necrosis of renal tubular cells after injury can release histones, ATP, and uric acid, which are known as DAMPs (77, 78). Then, DAMPs act on renal parenchymal cells and mesenchymal dendritic cells to induce an array of pattern recognition receptors, such as Toll-like receptors (TLRs) (79, 80). The subsequent innate immune response caused by TLR-4 initiates a variety of immune cells infiltrating into the kidney, promoting an inflammatory response and resulting in tubular cell death (81). After the onset of AKI, TLR4 signaling stimulates the production of IL-22, which is mainly sourced from intrarenal mononuclear phagocytes. IL-22-mediated regenerative functions on renal TECs are followed by the phosphorylation of JAK-STAT3 and ERK1/2 (50). This process increases endogenous TLR2 agonists and induces renal tubular progenitor cells to secrete regenerative proteins and microRNAs and prompt themselves to differentiate into renal TECs. Neutralizing endogenous IL-22 with IL-22 antibodies showed elevated indicators of kidney injury, such as kidney injury molecule (Kim)-1, π-glutathione S-transferase (GST), and α-GST, suggesting impaired tubular epithelial repair in AKI. In addition, IL-22 increased the survival of these epithelial progenitor cells and synthetically achieved renal epithelial regeneration. Overall, IL-22 promotes renal regeneration through epithelial cell proliferation as one of several promising strategies for treating (or avoiding) end-stage renal diseases (12).

Among the multiple causes of AKI, such as renal hypoperfusion, ischemia and toxicity, renal ischemia/reperfusion (I/R) is common, especially in proximal tubular cells (82). Studies have shown that renal dysfunction after I/R is partly attributed to cell apoptosis (83), and IL-22 has anti-apoptotic effects, with a possible molecular mechanism of activating AKT and inhibiting the mitochondrial apoptotic pathway, which is accompanied by an increase in Bcl-2 and a decrease in Bad (53). Compared to wild-type (WT) mice, IL-22-knockout (KO) mice had higher levels of urea nitrogen, and the survival rate at 7 days after injury was significantly lower, suggesting that IL-22 has protective effects on the kidney (53). The results of TUNEL staining displayed that the apoptotic tubule cells in the mIL-22-treated I/R group was significantly reduced; correspondingly, the levels of pAkt473, pSTAT3 and Bcl-2 were increased, and the renal cortex increased significantly, but WT mice had the opposite effects (53). In summary, activation of STAT3 and AKT occurred in the proximal TECs of mice after I/R, followed by an increase in Bcl-2 levels and a decrease in Bad levels, which may play a crucial role in IL-22-mediated regeneration and repair of epithelial cells in response to I/R-induced renal injury.

Apart from AKI, IL-22 is also a regenerative factor that plays an important role in the unilateral ureteral obstruction (UUO) model of chronic progressive obstructive nephropathy (55). In UUO, IL-22^+^ cells accumulate more abundantly in the renal interstitium than other cells. *IL-22^-/-^* mice showed significant tubular cell death and tubular atrophy compared with *IL-22^+/+^* mice. *In vitro* experiments indicated that IL-22 supplementation promoted the migration, proliferation, metabolic activity, and regeneration of human tubular epithelial cells. Specifically, the regenerative function of IL-22 on RTECs in chronic diseases was linked to the transduction of STAT-AKT-dependent pathways and consequent decrease of Bad protein that is known as a pro-apoptotic mediator. It can be hypothesized that the mechanism by which IL-22 mediates STAT-JAK pathway-induced cell regeneration may be associated with improving apoptotic events. Studies have shown that AKT activation ameliorates renal injury (84, 85). Erlotinib improved animal phenotypes by acting on the AKT signaling pathway and attenuating the activation of thylakoid cells and macrophages in a rat model of CKD (55). Considering the protective role of IL-22 in UUO, modulating IL-22 may be a potential therapeutic strategy in other forms of CKD.

### The Protective Role in Inhibiting Oxidative Stress

The pathogenesis of AKI is complex and diverse and includes oxidative stress, inflammation, and vascular injury, among which oxidative stress is considered a central exacerbating factor (86). It has been reported that mitochondria are easily damaged during the development of AKI and are the main intracellular source of reactive oxygen species (ROS) (87). ROS are highly reactive products that trigger apoptosis, necrosis, oxidative stress, and even induce chronic inflammation and renal fibrosis (88). Therefore, ROS are critical targets in oxidative stress-mediated AKI treatment, and ROS clearance might reshape the microenvironment of kidney (**Figure 2C**).

IL-22, a regenerative factor for RTECs, promotes regeneration and repair of renal tissue by inhibiting oxidative stress. *In vitro* IL-22 can ameliorate the accumulation of dysfunctional mitochondria in TECs after cisplatin injury by activating mitochondrial phagocytosis (89). Meanwhile, compared to the model group, IL-22 treatment protected the mitochondrial health of damaged RTECs, as evidenced by reductions in mitochondrial mass, mitochondrial ROS, and mitochondrial dysfunction. Mechanistically, IL-22 may reprogram PFBFK3 and activate STAT3-AMPK/AKT signaling, alleviating the accumulation of ROS and mitochondrial dysfunction in the kidney (90). Further, the reduction of ROS can ensure metabolic homeostasis of renal cells to some extent by downregulating the NLRP3/Caspase-1/IL-1β pathway, which protects renal function and promotes renal epithelial tissue regeneration and repair (54).

Other than that, once oxidative stress is initiated, persistent stimulation by excess ROS is an important link in the progression of kidney disease to fibrosis. This process recruits immune cells such as Th1, Th2, Th17, and regulatory T (Treg), which can release various growth factors, including IL-13 or TGF-β, promoting the excessive deposition of extracellular matrix and ultimately leading to renal fibrosis. The most common causes of CKD and renal fibrosis are diabetes mellitus (DM) (91–93), adult hypertension and childhood obstructive nephropathy (94). In a mouse model of chronic diabetic nephropathy, IL-22 reduced the abnormal deposition of renal collagen and extracellular matrix and therefore improved kidney fibrosis. The possible mechanism is mainly attributed to the inhibition of renal oxidative stress and mitochondrial dysfunction by IL-22 to improve the inflammatory response (51, 52).

## Pathological Effects of IL-22 in the Kidney

### The Pathological Effect in Aggravating Inflammatory Damage to the Kidney

Sustained kidney inflammation is a central player in the development of kidney diseases including IgAN, LN, and psoriatic kidney injury (57, 58, 95–98). Chemokines and their receptors play pivotal roles in inflammation by upregulating the migration and retention of circulating immune cells. Among these chemokines, high expression of CCL2 and CXCL10 was shown to play an important role in exacerbating proteinuria, deteriorating renal pathology and renal dysfunction in patients with LN. *In vitro* stimulation with rIL-22 alone was sufficient to upregulate the expression of CXCL10 and CCL2 in both primary kidney epithelial cells and a human kidney cell line (HK2), whereas increased macrophage infiltration was observed in the rIL-22-stimulated group. Furthermore, the expression levels of several kinds of chemokines, including CCL2 and CXCL10, have been found to be significantly reduced in rIL-22-deficient and IL-22R-deficient MRL/lpr mice, accompanied by reduced kidney injury in mice. Hu *et al.* found that IL-22 activated the STAT3 signaling pathway, thus causing deteriorating LN in lupus-prone mice. IL-22 deficiency ameliorated proteinuria, renal function and pathological impairment through downregulating the expression of CCL2 and CXCL10 and reducing the filtration of macrophages into the kidney, ultimately decreasing the systematic disease and the severity of LN (14). These findings indicated that IL-22 might play a role in aggravating inflammatory damage to the kidney and provide a promising novel therapeutic target (14) (**Figure 2D**).

In addition, IL-22 can also induce the inflammation in the kidney by affecting the complement system. The complement system serves as a vital part of the innate immune system that fights against invasive pathogens; however, uncontrolled activation of complement also promotes excessive inflammation and injury in various kidney diseases, such as LN and IgAN (99, 100). The wide variety of kidney diseases related to complement activation might be attributed to glomerular endothelial cell fenestrations and deficiencies in intrinsic complement regulators in the glomerular basement membrane (101). Indeed, the deposition of complement components in glomeruli has been commonly observed in IgAN and LN patients (102, 103). The association of IL-22 with the complement system was uncovered by analyzing the Gene Expression Omnibus dataset, which showed that treatment with IL-22 positively correlated with increased levels of C3 (56). This finding was further supported by the phenomenon that the mRNA level of C3 was reduced in the liver tissue of IL-22^-/-^ mice after *Clostridium difficile* infection, and this effect was reversed by systemic administration of IL-22. The mechanism by which IL-22 enhances C3 expression has not been entirely elucidated, but treatment with a STAT3 inhibitor reverses IL-22-induced C3 upregulation, suggesting that the STAT3 signaling pathway may be involved in IL-22-induced upregulation of complement C3 (104). The upregulation of C3 induced by IL-22 has also been demonstrated to exacerbate kidney injury. IL-22 or IL-22R deficiency decreases the deposition of C3 in the kidney but increases serum C3 levels, thus contributing to significant alleviation of kidney injury and prolonged survival rates in the murine model of LN, which is characterized by lower serum C3 levels and higher C3 deposition in the kidney (14). These findings suggested that genetic or pharmaceutical modification to dampen IL-22 signaling may be at least partially alleviate the kidney inflammation in patients with IgAN and LN by downregulating the complement pathway.

### The Pathological Effect in Inducing Chronic Renal Anemia

Anemia is a common complication of chronic kidney disease (CKD) that can increase the risks of cardiovascular diseases (59). The reasons for anemia were previously thought to be deficient erythropoietin production and poor iron uptake in CKD patients, but therapies intended to increase erythropoietin and iron levels were only effective in a minority of patients (59). Recent progress has been made in identifying a novel mechanism of IL-22 underlying the development of anemia in CKD patients (**Figure 2E**).

While IL-22 is widely expressed by innate immune cells, the IL-22R was unexpectedly discovered on erythroid precursors, and this receptor was involved in inducing apoptosis in erythroid precursors to exacerbate anemia in CKD patients (60, 61). Mice deficient in IL-22 or IL-22RA1 on a right open reading frame 2 (Riok2)-haploinsufficient background develop excess red blood cells (RBCs) and exhibit downregulated apoptosis in erythroid precursors, while exogenous supplementation with rIL-22 exacerbates phenylhydrazine-induced anemia, as evidenced by upregulated apoptosis and reduced numbers of RBCs. Importantly, rIL-22 treatment failed to worsen phenylhydrazine-induced anemia in mice with erythroid cell-specific knockout of IL-22RA1, suggesting that IL-22RA1 expression on erythroid precursors is absolutely involved in the induction of erythroid precursor apoptosis. Further experiments have identified that p53 is upregulated by IL-22 treatment *in vitro* and in IL-22RA1+ erythroid precursors from Riok2-haploinsufficient mice. Pharmaceutical inhibitor of p53 suppressed apoptosis in IL-22-free media, indicating that p53 is a critical interrelated molecule by which IL-22 induces apoptosis in erythroid precursors, thus leading to the exacerbation of anemia.

Beyond inducing apoptosis in erythroid progenitors, IL-22 has also been identified to be a positive regulator of hepcidin, which is a central mediator of iron homeostasis. A study showed a marked increase in hepcidin in hepatoma cells treated with IL-22 (105). Mice deficient in IL-22 have decreased transcriptional levels of the hepatic hepcidin 1 and pro-hepcidin gene in response to LPS, while treatment with the IL-22-Fc fusion protein leads to increased levels of hepcidin and iron deficiency *in vivo*. The mechanism by which IL-22 enhances hepcidin production is not completely understood, but the Tyk2 signaling pathway is likely involved, as IL-22-induced hepcidin upregulation and hemoglobin downregulation are impaired in Tyk2-deficient mice (106). Moreover, plasma levels of IL-22 are significantly elevated in CKD patients with anemia, and this increase correlates with decreased concentrations of hemoglobin (107). These findings highlight the importance of IL-22 in exacerbating anemia by inducing hepcidin production and apoptosis, which may hopefully provide a new target for the treatment of anemia in CKD patients.

### The Possible Pathogenic Effect in Vascular Inflammatory Diseases-Related Renal Injury

Microscopic polyangiitis (MPA) and Granulomatosis with polyangiitis (GPA) are chronic autoimmune inflammatory vascular lesions with unknown etiology, and renal involvement is a typical and potentially life-threatening symptom in these diseases (108). To date, IL-22 has been poorly studied in renal damage associated with these vascular inflammatory diseases. However, high pathogenicity of Th17 to the kidney was observed in GPA patients positive for anti-neutrophil cytoplasmic antibodies (2, 109). The possible mechanism explored was that early infiltration of Th17 cells leads to the upregulation of CXCL9, which subsequently stimulates renal cells to express glutamate-leucine-arginine motif (ELR) chemokines (CXCL1, 2, and 5) that recruit neutrophils to the kidney. And IL-22, the most marked cytokine of Th17 cells, had the possibility of being involved in their pathogenic role in renal disease (110). It was also confirmed that specific IL-22 expression was detected in the renal tissues of GPA patients and diffuse IL-22^+^ cell infiltration was found in the renal interstitium and local glomeruli medially in renal tissue biopsies, suggesting a possible pathogenic role of IL-22 in GPA-related renal injury (111). Meanwhile, another group found that after 6 months of follow-up of GPA, IL-22 was significantly reduced with the restoration of the kidney and other organs (108). Therefore, the proposed therapeutic strategies targeting Th17 cells, and the corresponding cytokine IL-22 may have promising applications for the treatment of vasculitis-associated nephropathy.

## Clinical Trials

Cytokine-targeted therapy is now a promising therapeutic strategy for various kidney diseases due to the side effects of steroid hormones and other immunosuppressive agents. Currently, no drugs that directly target IL-22 have been approved by the FDA for the treatment of kidney disease. As for other diseases, IL-22-related drugs, including IL-22-Fc, IL-22 specific antibodies and inhibitors, and IL-22R inhibitors, have been in clinical trials for different periods. (**Table 2**).

**Table 2:** Clinical Trials Related to IL-22 Based on Clinicaltrials.gov.

Agent	Indication	Identifier	Mechanism	Disease (Clinical Phase)	Sponsor	References
**ILV-094**	antibody to neutralize IL-22	NCT00883896	Inhibition of inflammatory response	Rheumatoid Arthritis (Phase-II)	Rockefeller University	(112)
**ILV-094**	antibody to neutralize IL-22	NCT01941537	Inhibition of epithelial proliferation	Atopic dermatitis (Phase-II)	Rockefeller University	(113)
**F-652**	IL-22 IgG2 Fc fusion protein	NCT02406651	Regulating T-lymphocyte function to stabilize the immune system	Acute Graft *vs* Host Disease (Phase I)	Generon (Shanghai) Corporation Ltd.	(114)
**F-652**	IL-22 IgG2 Fc fusion protein	NCT04498377	Inhibition of inflammatory response	moderate to severe COVID-19(Phase-II)	Generon (Shanghai) Corporation Ltd.	(115)
**IL-22BP**	IL-22 binding protein	NCT02847052	Inhibition of continuous inflammatory and decreasing the risk of cancer	Inflammatory Bowel Disease (Complete)	Nantes University Hospital	(116)
**IL-22BP**	IL-22 binding protein	NCT04130189	Inhibition of epithelial proliferation	Atopic Dermatitis (Not Applicable)	Nantes University Hospital	(117)
**IL-22**	cytokine	NCT01918462	up-regulating several antioxidant, antiapoptotic, and antimicrobial genes of liver cells	Alcoholic Hepatitis (Complete)	University of Aarhus	(118)

The IL-22-neutralizing antibody Fezakinumab (ILV-094) has currently finished phase I and II clinical trials for rheumatoid arthritis and atopic dermatitis, respectively (4, 112). The only reported adverse reactions with a high incidence were upper respiratory tract infections of different pathogens, including viruses and fungi. F-652, a fusion protein of IL-22 fused to the IgG2 anchoring region, is used its tissue-protective and regenerative effects to treat graft-versus-host disease (aGVHD) and severe COVID-19 (113, 114). After that, existing clinical studies have assessed the expression of IL-22BP in skin and serum to deeply explore the protective role of IL-22BP in atopic dermatitis and inflammatory bowel disease (115, 116). In addition, the IL-22-related signaling pathways PGE2/IL22/IL17 and IL-17/IL-22 are receiving increasing attention in eczema, psoriasis, and chronic obstructive pulmonary disease (117, 119). The production and effects of the cytokine IL-22 in patients with alcoholic hepatitis has been the research interests by certain research teams (120). However, some studies have shown that IL-22 can promote the progression of existing tumors, especially colon cancer, by activating STAT3-related signaling pathways (118, 121). Therefore, the risk of concomitant tumor exacerbation needs to be carefully assessed when applying recombinant IL-22.

In the treatment of renal diseases, the application of enhancing or attenuating IL-22 effects should be determined according to the specific microenvironment. Because IL-22 is able to protect against pathogenic bacterial infections, promote cell proliferation, inhibit apoptosis, and suppress oxidative stress, we propose an attempt to apply IL-22 or its fusion proteins in infectious kidney diseases and AKI. Considering the role of IL-22 in activating the complement system and recruiting chemokines, IL-22-neutralizing antibody and IL-22BP may be used to treat immune-related renal diseases such as IgAN and LN.

## Conclusion

In conclusion, IL-22 is a double-edged sword for the kidney. On one hand, studies in diabetic nephropathy, obstructive nephropathy, and acute kidney injury models indicate that IL-22 plays a vital role in tissue protection and regenerative repair. This cytokine is essential not only in host defense against various pathogens but also in restoring immune quiescence and tissue homeostasis after an inflammatory response. Additionally, IL-22 exerts pathogenic effects in certain renal diseases, including systemic lupus erythematosus, IgA nephropathy, and anemia induced by CKD, due to recruitment of chemokines and uncontrolled activation of complement. Upon the protective and pathogenic roles of IL-22 in the kidney, the application of IL-22 in the treatment of renal diseases can be attempted. Further research on IL-22 biology will undoubtedly contribute to a better understanding of many kidney diseases and potential therapeutic approaches, opening the way for the identification of new therapeutic approaches and directions for the application in the clinic.

## Author Contributions

QM and JL contributed equally to this paper. QM, JL, and CX conceived and drafted the paper. YB and FL prepared the tables and figures. BC was involved in the compilation of the references. DJ and HX instructed and revised the manuscript. All authors contributed to the article and approved the submitted version.

## Funding

This research was funded by the National Natural Science Foundation of China under grant 82170793 and The Collaborative Research Projects of Greater Bay Area Institute of Precision Medicine (Guangzhou) in 2021 (IPM2021C003).

## Conflict of Interest

The authors declare that the research was conducted in the absence of any commercial or financial relationships that could be construed as a potential conflict of interest.

## Publisher’s Note

All claims expressed in this article are solely those of the authors and do not necessarily represent those of their affiliated organizations, or those of the publisher, the editors and the reviewers. Any product that may be evaluated in this article, or claim that may be made by its manufacturer, is not guaranteed or endorsed by the publisher.
